# Effects on tumor immunity of anti-TGF-beta with different isoform specificities

**DOI:** 10.1186/2051-1426-2-S3-P62

**Published:** 2014-11-06

**Authors:** Masaki Terabe, Faith C Robertson, Shingo Kato, Emma De Ravin, Amer Mirza, Jay A Berzofsky

**Affiliations:** 1 NCI/NIH, Bethesda, MD, USA; 2 Xoma Corp, Berkeley, CA, USA

## 

TGF-beta has been shown to play critical roles in immune suppression that occurs in cancer patients. TGF-beta has three isoforms, TGF-beta1, 2 and 3. We and others have reported that blockade of all three isoforms enhances tumor immunosurveillance and cancer vaccine efficacy in mouse tumor models. Among the three isoforms of TGF-beta, TGF-beta1 and TGF-beta2, especially TGF-beta1, have been widely studied for their suppressive activity on T cell activation as well as their ability to induce immune suppressive cells (e.g. Treg and MDSC). However, very little is known about the function of TGF-beta3 in either immunology or cancer although there are some studies that suggest this isoform may have some beneficial activity for cancer patients. Thus, in this study, we asked whether it is necessary to block TGF-beta3 to enhance tumor immunity by using monoclonal antibodies with different specificities against TGF-beta isoforms. In a pulmonary metastasis model of colon carcinoma CT26, blockade solely of TGF-beta1 and TGF-beta2 but not TGF-beta3 significantly reduced tumor burden to a degree similar to that achieved by blockade of all isoforms, suggesting that it is not necessary to block TGF-beta3 to facilitate tumor immunosurveillance. In a subcutaneous TC-1 model in which a peptide based vaccine was administered to mice with well established tumors (at least 5 mm in diameter), TGF-beta blockade significantly enhanced vaccine efficacy to reduce tumor size although blockade of TGF-beta alone had no effect on tumor growth. Since blockade of only TGF-beta1 and TGF-beta2 significantly improved vaccine efficacy similarly to blockade of all isoforms, we concluded that inhibition of TGF-beta3 is not necessary to enhance the therapeutic tumor vaccine efficacy. Surprisingly blockade of TGF-beta did not change the number of MDSCs, Th17 or Treg cells in tumors. It also did not change the number of vaccine specific-CD8^+ ^T cells. However, it increased the number of T-bet expressing CD4^+ ^and CD8^+ ^T cells, suggesting that more Th1-type T cells infiltrated into tumors when TGF-beta was blocked. In conclusion, blockade of TGF-beta1 and TGF-beta2 is sufficient to enhance tumor immunity, and sparing TGF-beta3 may be beneficial for patients.

